# Discovery of novel and highly potent small molecule inhibitors targeting FLT3-ITD for the treatment of acute myeloid leukemia using structure-based virtual screening and biological evaluation

**DOI:** 10.3389/fphar.2025.1511257

**Published:** 2025-02-03

**Authors:** Kun Shi, Ye Hong, Huajing Liu, Xiaotian Yang, Fengzhen Wang, Yanming Zhang

**Affiliations:** ^1^ Clinical Medical College, Xuzhou Medical University, Xuzhou, China; ^2^ Department of Orthopedics, Xuzhou Central Hospital, Xuzhou Clinical School of Xuzhou Medical University, Xuzhou, China; ^3^ Department of Hematology, The Affiliated Huai’an Hospital of Xuzhou Medical University, Huai’an, Jiangsu, China; ^4^ Department of Hematology, Binhai County People’s Hospital, Yancheng, Jiangsu, China; ^5^ Jiangsu Key Laboratory of New Drug Research and Clinical Pharmacy, Xuzhou Medical University, Xuzhou, Jiangsu, China; ^6^ Department of Pharmacy, Suining People’s Hospital Affiliated to Xuzhou Medical University, Suining, China

**Keywords:** FLT3-ITD mutation, acute myeloid leukemia (AML), small molecule inhibitor, structure-based virtual screening, biological evaluation

## Abstract

Considering the essential role of FLT3-ITD mutations in the development of acute myeloid leukemia (AML), the research and development of FLT3 inhibitors hold significant therapeutic potential. In this study, we identified a novel, highly potent small molecule inhibitor, FLIN-4, targeting FLT3 through structure-based virtual screening. Notably, FLIN-4 showed exceptional inhibitory effects in kinase activity inhibition assays, exhibiting a potent inhibitory effect against FLT3 (IC_50_ = 1.07 ± 0.04 nM). This potency was significantly superior to that of the known positive inhibitor Midostaurin, showing approximately 27 times higher inhibitory potency. Molecular dynamics simulations have confirmed the stable interaction between FLIN-4 and FLT3. Furthermore, cytotoxicity assays revealed that FLIN-4 has significant anti-proliferative activity against the AML cell line MV4-11 (IC_50_ = 1.31 ± 0.06 nM). Overall, these data suggest that FLIN-4, as a potential therapeutic candidate for AML, is valuable for further research and development.

## 1 Introduction

Acute myeloid leukemia (AML) represents a prevalent malignancy in the adult population, distinguished by the accelerated growth of aberrant myeloblasts within the hematopoietic tissue of the bone marrow. These immature and dysfunctional cells fail to effectively perform immune functions (Siegel et al., 2024). The incidence and mortality rates of AML are significant in the spectrum of hematological malignancies, accounting for approximately 1% of all new cancer cases (Siegel et al., 2024; Joshi et al., 2024). With the aging of the global population, the incidence of age-related cancers, including AML, is on the rise (Anderson et al., 2024). Strategies for managing Acute Myeloid Leukemia (AML) commonly encompass chemotherapy, targeted therapeutic interventions, and the transplantation of hematopoietic stem cells (Kantarjian et al., 2021). Chemotherapy drugs, while killing leukemia cells, can also affect the function of normal cells, leading to severe side effects (Li et al., 2023). Moreover, hematopoietic stem cell transplantation is a high-risk treatment that may result in serious complications such as organ damage and infections (Young et al., 2021). Hence, there is a pressing requirement for the development of novel and efficacious therapeutic approaches for the management of AML.

As a receptor tyrosine kinase, FMS-like tyrosine kinase-3 (FLT3) exerts a critical regulatory influence on the proliferation and differentiation processes of hematopoietic stem cells (Daver et al., 2019; Novatcheva et al., 2022). When FLT3 binds to its specific extracellular ligand, it triggers a series of molecular events that include receptor dimerization, self-phosphorylation, and the activation of multiple downstream signaling cascades, such as the RAS/MAPK, JAK/STAT5, and PI3K/AKT pathways (Antar et al., 2020; Griffith et al., 2004; Takahashi, 2011). By coordinating their actions, these pathways contribute to the proliferation and differentiation of myeloid lineage cells. In AML, genetic mutations in the FLT3 gene are prevalent and are typically linked to unfavorable outcomes in myeloproliferative diseases (Kennedy and Smith, 2020; Kiyoi et al., 2020; Kawashima, 2021). Within the cohort of AML patients, a prevalence of roughly 30% is attributed to FLT3 mutations, with internal tandem duplications (ITD) being the most frequently observed subtype (Castaño-Bonilla et al., 2021). FLT3-ITD mutations are in-frame duplications of variable length and position within the juxtamembrane domain of the receptor. In wild-type (WT) FLT3, the juxtamembrane domain inhibits receptor activation. However, the presence of an ITD disrupts this inhibition, leading to constitutive kinase activation. As a consequence, the PI3K/AKT and ERK/MAPK signaling cascades, which are instrumental in driving cell growth, are excessively stimulated. Additionally, the JAK/STAT5 pathway is also hyperactivated. These activations collectively lead to an enhancement of cancer cell replication and a suppression of apoptosis (Daver et al., 2019; Daver et al., 2021; Nogami et al., 2015). Therefore, FLT3 is considered a potential therapeutic target for the treatment of AML.

FLT3 inhibitors exert their action by competitively inhibiting the ATP-binding site within the FLT3 receptor, leading to cell cycle arrest and differentiation (Sung et al., 2024; Zhao et al., 2022). A variety of FLT3 inhibitors are currently in clinical use or under investigation, including Gilteritinib, Quizartinib, Midostaurin, and Sorafenib (Abbas et al., 2019; Cortes et al., 2018; Tang et al., 2020; Marjoncu and Andrick, 2020). Gilteritinib is a second-generation FLT3 inhibitor classified as an ATP-competitive type I inhibitor (James et al., 2020). It curbs the expansion and endurance of malignant cells by impeding the FLT3-ITD mutation, thereby preventing the activation of downstream signaling pathways. It has received regulatory approval for use in patients exhibiting FLT3-mutated AML, specifically those with disease that has recurred or is non-responsive to prior treatments, and it has demonstrated good efficacy and tolerability in clinical trials (Yuan et al., 2022). Quizartinib has been approved in Japan for the treatment of patients with relapsed or refractory FLT3-mutated AML (Takahashi et al., 2019; Usuki et al., 2019), and it has shown good efficacy in clinical trials even at low doses (Fletcher et al., 2020). Although these FLT3 inhibitors have shown excellent therapeutic effects, challenges such as low efficacy, low selectivity, and resistance remain obstacles in the treatment of AML patients (Swaminathan et al., 2021). Overall, the development of novel and potent FLT3 inhibitors continues to represent a significant challenge in the field.

Structure-based virtual screening, a computational method for drug lead identification, has emerged as a more efficient and cost-effective alternative to conventional high-throughput screening approaches (Sadybekov and Katritch, 2023). In our research, we employed this method to successfully uncover a novel FLT3 inhibitor, designated as FLIN-4. Subsequent cytotoxicity assays confirmed the substantial inhibitory effect of FLIN-4 on cell proliferation. Moreover, molecular dynamics simulations were conducted to evaluate the stability of the FLIN-4 and FLT3 interaction, further validating the robustness of this interaction. Our findings suggest that the rational design of FLT3 inhibitors through structure-based virtual screening is a viable strategy, potentially contributing to advancements in AML therapy. This work is anticipated to offer insightful direction for the future development of potent FLT3 inhibitors.

## 2 Materials and methods

### 2.1 Reagents

The human AML cell line MV4-11, which bears exclusive FLT3-ITD mutation genes, was purchased from the American Type Culture Collection (ATCC, Manassas, VA, United States), and the AML cell line NB4, a FLT3-wildtype cell line, was obtained from the China Center for Type Culture Collection (CCTCC, Wuhan, China). In addition, the normal hematopoietic cell line was COLO 829BL from ATCC. The cells were maintained in RPMI-1640 medium supplemented with 10% FBS, and 1% penicillin and streptomycin. The cells were cultured in an incubator at 37°C with 5% CO_2_. All compounds, sourced from WuXi AppTec (Shanghai, China), exhibit a purity level exceeding 96%, with their respective lot numbers detailed in Supplementary Table S1. The high-performance liquid chromatography (HPLC) spectra data for each compound are provided in the (Supplementary Figures S1–S6). The FLT3-ITD mutant protein was purchased from Abcam (Cambridge, United Kingdom).

### 2.2 Virtual screening

The crystal structure of FMS-like tyrosine kinase-3 (PDB ID: 6JQR) was obtained from the Protein Data Bank (PDB). The PDB file of the FLT3 protein crystal structure was imported into the MOE software. The protein structure was preprocessed using the Quickprep tool in MOE, which included the removal of unbound water, the addition of polar hydrogens, and energy minimization.

The QuaSAR-CombiGen tool of MOE was used to constructed a library of 35,000 compounds. Attachment ports are terminal atoms named “An,” where n is a positive integer. The carbon atom on the terminal end of each scaffold molecule is labeled as “A1” port. The atom on the terminal end of each chemical group is labeled as “A0” port. Using QuaSAR-CombiGen tool, the entire combinatorial library was enumerated by exhaustively cycling through all combinations of 70 scaffold molecules at attachment “A1” port and 500 chemical groups at attachment “A0” port. According to this method, a complete enumerated library of 35,000 combinatorial compounds was generated using QuaSAR_CombiGen. Finally, the enumerated library was written to an output database containing 35,000 compounds. The energy minimization algorithm of MOE was further used to convert 2D-structures of compounds in database to 3D-structures.

Based on the processed crystal structure of FLT3 protein, the Dock tool in MOE was utilized to dock the 35,000 compounds at the active binding site of the FLT3 protein. The molecular docking algorithm used the triangular matching method and the London dG scoring algorithm, and a docking score threshold was set to identify potential compounds. Compounds with docking scores below the reasonable threshold were selected as potential FLT3-targeting compounds.

### 2.3 Microscale thermophoresis (MST)

MST was employed to determine the binding affinity between test compounds and FLT3-ITD in a buffer solution containing 50 mM Tris and 230 mM NaCl at pH 7.0. The protein was labeled with a fluorescence tag utilizing a Lys Labeling Kit (NanoTemper), with a final concentration of 50 nM being achieved. Adhering to the manufacturer’s guidelines, the compounds were titrated from an initial concentration of 12.5 μM with a 1:1 dilution ratio. Next, each sample was centrifuged at 15,000 rpm for 5 min, followed by loading into standard glass capillaries for MST analysis.

### 2.4 Kinase-inhibition assay

The inhibitory rates of FLINs 1-6 against FLT3-ITD were assessed utilizing the ADP-Glo™ Kinase Assay Kit (Promega, Madison, WI) (Wei et al., 2024). The concentrations used were as follows: FLT3-ITD at 10 ng/μL, substrate at 0.1 mg/mL, and ATP at a dose of 100 μM (FLT3-ITD). The test compounds or Midostaurin, kinase, substrate, and ATP were diluted to the specified concentrations in kinase buffer and incubated for 30 min. Subsequently, the kinase detection reagent was added and incubated for an additional 15 min. Finally, the mixture was incubated for 1 h, and relative luminescence units (RLU) were measured.

### 2.5 Kinase selectivity assays

Following the standardized kinase activity assay protocol as previously reported, we utilized the SelectScreen Kinase Profiling Service (Thermo Fisher Scientific) to determine the kinase selectivity profile of FLIN-4 (van der Wel et al., 2020). Through a ten-dose dilution series ranging from 0.25 μM to 128 μM in dose-response experiments, we precisely measured the inhibitory effect of FLIN-4 on the substrate phosphorylation reactions catalyzed by a panel of kinases, and thus ascertained the half-maximal inhibitory concentrations (IC_50_) for each kinase.

### 2.6 Molecular dynamics (MD) simulation

The crystal structure of FLT3 (PDB ID: 6JQR) was obtained from the PDB. The MOE software was then employed to model the crystal structures of FLT3 in complex with the ligands FLIN-3 and FLIN-4. To investigate the dynamic behavior of these complexes, we performed molecular dynamics (MD) simulations using GROMACS software, version 2022. The topological structure of the FLT3 protein was constructed under periodic boundary conditions, employing the AMBER99SB-ILDN force field. The topology file for FLT3 was generated using the Acpype Server (www.bio2byte.be). Following this, the ligand’s parameters were integrated with the protein’s parameters to form the complete complex system. The complex system was dissolved in a cubic box with an edge length of 1.0 nm, which was also filled with the SPC water model. In the entire simulation system, equilibrium ions Na^+^ and Cl^−^ were added to neutralize the complex system. Next, the system energy was minimized using the steepest descent algorithm with 5,000 steps. Subsequently, 100 ps of NVT equilibrium and 100 ps of NPT equilibrium were performed, maintaining the experimental conditions at a constant temperature (300 K) and constant pressure (1 bar). Eventually, a 50 ns molecular dynamics simulation was conducted on the complex system, and trajectory data was recorded at intervals of 10 ps. According to the results, stability was evaluated in conjunction with RMSD, RMSF, the secondary structure of the protein, and the protein’s radius of gyration.

### 2.7 MTT assay

The effects of the test compounds on MV4-11, NB4, and COLO 829BL cell lines were quantitatively evaluated according to the methods previously reported (Zhou et al., 2022). Finally, nonlinear regression was used to plot the dose-response curve and to determine the IC_50_ values, with these data being analyzed using Prism GraphPad 10.0 software.

### 2.8 Western blot assay

The MV4-11 cell line was subjected to a 4 h exposure to varying concentrations of the FLIN-4 compound. Subsequent to this treatment, cellular lysis was conducted using RIPA lysis buffer (Sigma-Aldrich, United States) to isolate total cellular proteins. Subsequently, protein concentrations were precisely quantified using the BCA protein assay kit (Beyotime, Nantong, China). Western blot analysis was carried out in accordance with established methodologies (Zhou et al., 2022). The primary antibodies utilized in this study included: rabbit anti-FLT3 (21049−1-AP, Proteintech Group), rabbit anti-phospho-FLT3 (ab171975, Abcam), rabbit anti-Stat5 (#94205, CST), rabbit anti-Phospho-Stat5 (#4322, CST), rabbit anti-ERK (16443−1-AP, Proteintech Group), rabbit anti-phospho-ERK (28733−1-AP, Proteintech Group), and a rabbit anti-GAPDH antibody (10494−1-AP, Proteintech Group) served as an endogenous loading control.

## 3 Results

### 3.1 Structure-based virtual screening of targeting FLT3

**FIGURE 1:**
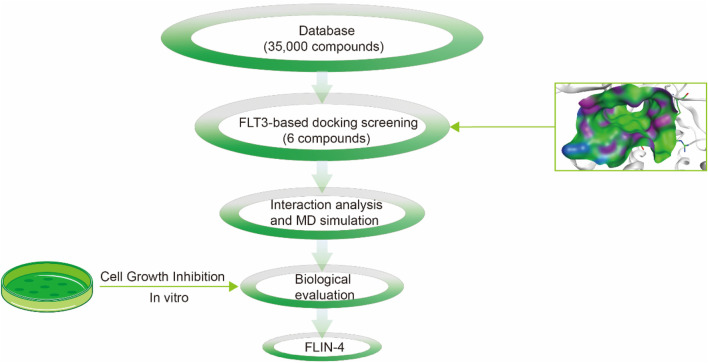
The workflow for the multi-step virtual screening of FLT3 inhibitors encompasses the processes from compound library screening, molecular docking, molecular dynamics simulation, biological evaluation, to the final identification of candidate compounds.

**FIGURE 2:**
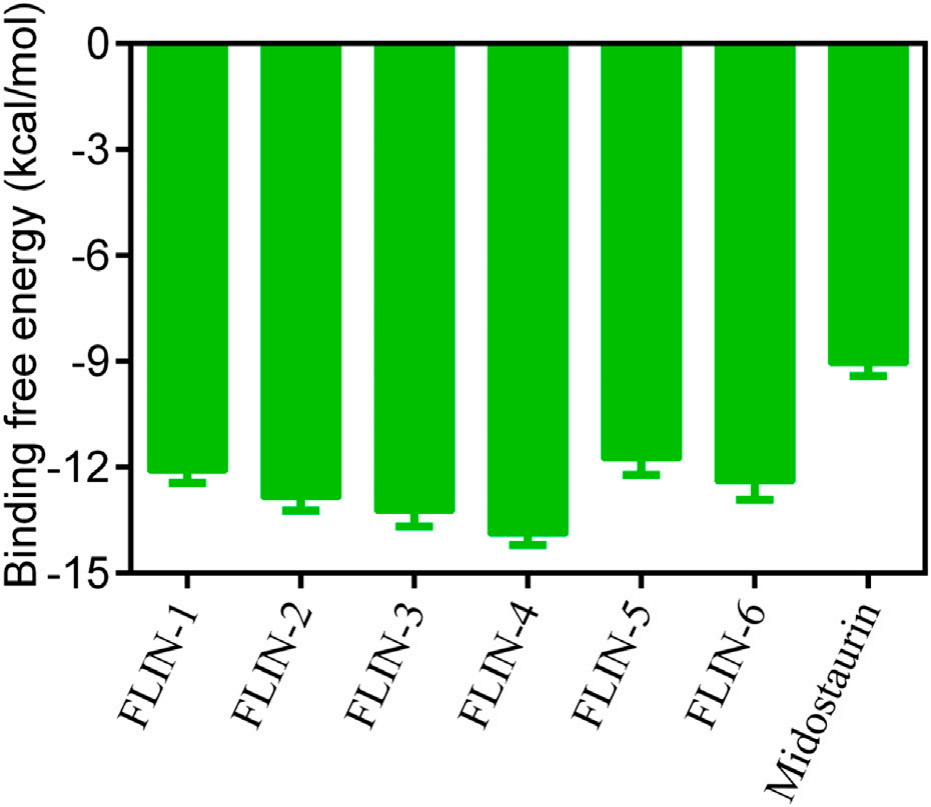
The binding free energy (kcal/mol) of FLINs 1–6, with lower values denoting higher affinity for FLT3 and implying a more potent inhibitory activity.

**FIGURE 3:**
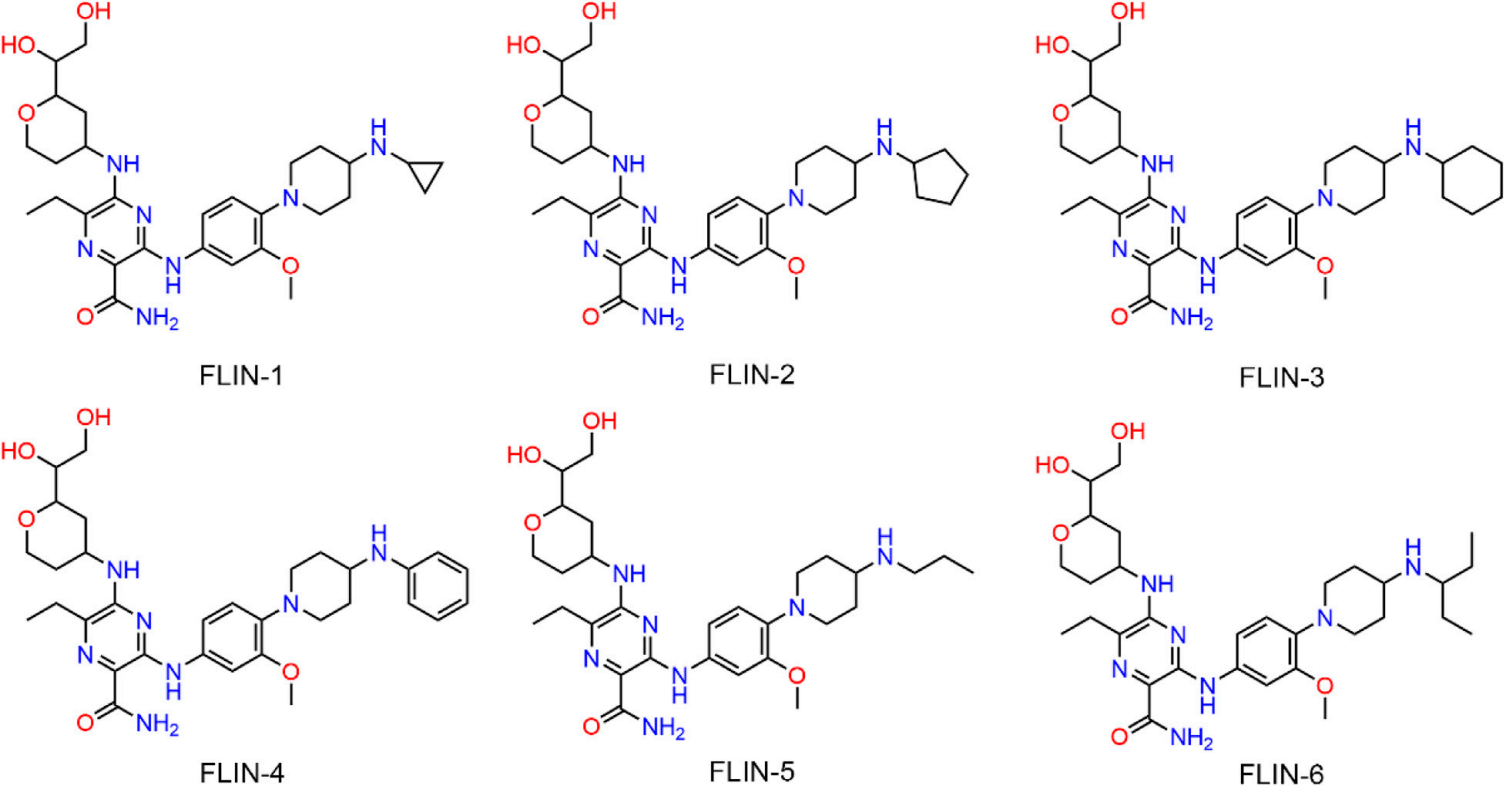
Depiction of the chemical architectures for FLINs 1–6.

The multi-step virtual screening workflow for FLT3 inhibitors is showed in Figure 1. Initially, a virtual 2D database containing 35,000 compounds was constructed using the Molecular Operating Environment (MOE) program. All compounds in the database were converted from 2D chemical structures to 3D structures using the MOE energy minimization algorithm. Subsequently, the crystal structure of the FLT3 protein was obtained from the PDB database and imported into the MOE software. The protein structure was preprocessed using the Quickprep tool in MOE, which included the removal of unbound water, the addition of polar hydrogens, and energy minimization. Based on the processed crystal structure of FLT3 protein, the Dock tool in MOE was utilized to dock the 35,000 compounds into the active site of the FLT3 protein. Moreover, the docking score was used to assess the binding affinity of the compounds with FLT3, where a lower docking score reflects a stronger binding affinity. Generally, the better the docking score, the lower the binding free energy, indicating a stronger binding affinity. Midostaurin was used as a positive control. Therefore, by setting a reasonable docking score threshold of less than −9.06 kcal/mol (referencing the docking score of Midostaurin), a total of 158 compounds were screened out. Ultimately, the top six compounds with the lowest binding free energy (referred to as FLINs 1–6) were selected for a detailed interaction analysis. The binding free energy of FLINs 1–6 is depicted in Figure 2. Among them, FLIN-4 exhibited the lowest binding free energy, which indicating that it has the strongest binding affinity to FLT3. The chemical structures of FLINs 1–6 are presented in Figure 3.

### 3.2 Interaction analysis

**FIGURE 4:**
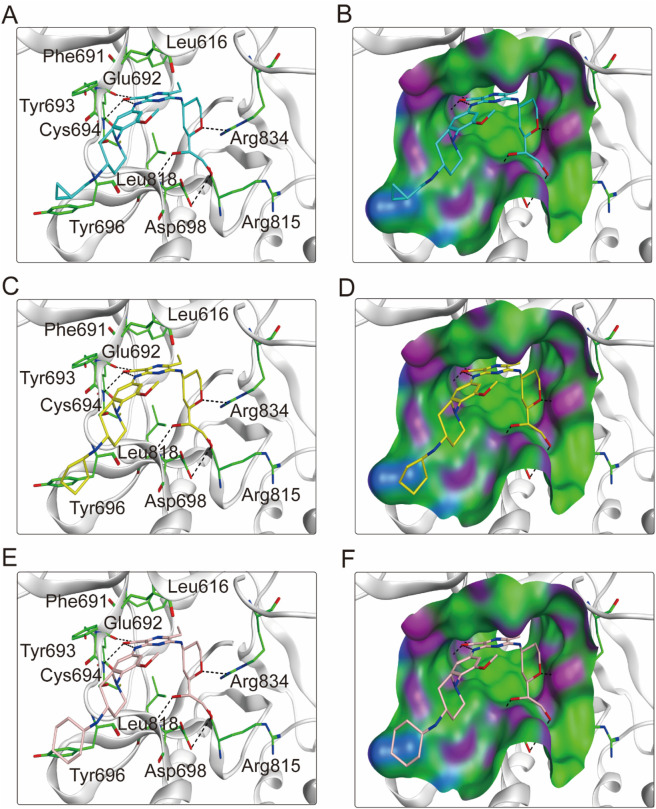
The binding modes of FLINs 1-3 to FLT3. **(A, B)** FLIN-1, cyan; **(C, D)** FLIN-2, yellow; **(E, F)** FLIN-3, pink. Residues of FLT3 in the binding surface are shown as green sticks. The hydrogen bonds are represented in black dashed lines. The pocket surfaces of the FLT3 are shown as H-bonding (purple), hydrophobicity (green), and mild polar (blue) regions.

**FIGURE 5:**
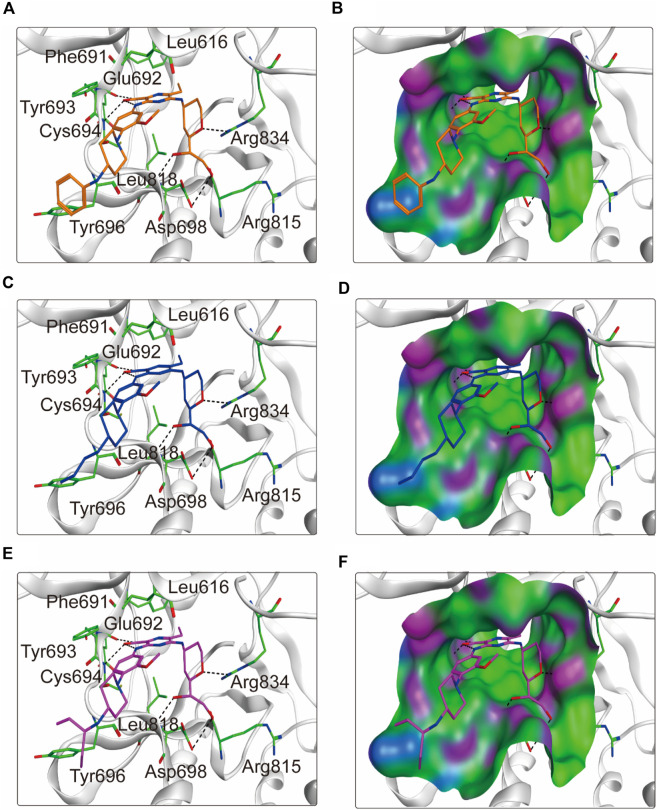
The binding modes of FLINs 4–6 to FLT3. **(A, B)** FLIN-4, orange; **(C, D)** FLIN-5, blue; **(E, F)** FLIN-6, purple. Residues of FLT3 in the binding surface are shown as green sticks. The hydrogen bonds are represented in black dashed lines. The pocket surfaces of the FLT3 are shown as H-bonding (purple), hydrophobicity (green), and mild polar (blue) regions.

To elucidate the binding mechanism of FLINs 1–6, we conducted a thorough analysis of their binding modes with FLT3. The predicted binding modes and binding surface diagrams of FLINs 1–3 and FLINs 4–6 are shown in Figures 4, 5, respectively. In FLINs 1–6, the oxygen atom of the amide on the benzene ring acted as a hydrogen bond acceptor, forming a hydrogen bond interaction with the nitrogen atom on the key residue Cys694. Moreover, the nitrogen atom of the amide on the benzene ring in FLINs 1–6 acted as a hydrogen bond donor, forming a hydrogen bond interaction with the oxygen atom on the carbonyl group of the key residue Glu692. The hydroxyl groups on FLINs 1–6 not only formed hydrogen bonds with the oxygen atoms on the carboxylate group of Asp698, but also another hydroxyl group on FLINs 1–6 formed hydrogen bonds with the nitrogen atom on Asp698. Moreover, the oxygen atoms in FLINs 1–6, acting as hydrogen bond donors, formed hydrogen bond interactions with the nitrogen atoms of the guanidinium group on Arg834. Additionally, the benzene rings on FLINs 1–6 formed π-π interactions with the benzene ring on Tyr693, which may have enhanced the stability of the binding between FLINs 1–6 and FLT3. Notably, the terminal benzene ring of FLIN-4 extended out to form π-π interactions with the benzene ring on Tyr696, which may help to enhance the binding affinity. Therefore, the previously mentioned binding modes indicated that FLINs 1–6 may have been stably bound within the active pocket of FLT3, with FLIN-4 potentially exhibiting a stronger binding affinity to FLT3.

### 3.3 MST assay

To validate the binding affinity of the screened FLINs 1-6 with FLT3-ITD, we employed MST to quantify the interactions between FLINs 1-6 and FLT3-ITD and to determine the equilibrium dissociation constants (*K*_d_). The results of the assay are presented in Supplementary Table S2. Compared to the positive control Midostaurin (*K*_d_ = 17.08 ± 3.14 nM), FLINs 1-6 exhibited stronger binding to FLT3-ITD (*K*_d_ = 0.95–12.47 nM), with FLIN-4 demonstrating the highest binding affinity (*K*_d_ = 0.95 ± 0.06 nM).

### 3.4 Comprehensive kinase inhibition analysis of FLIN-4

We conducted kinase activity inhibition assays to evaluate the inhibitory effect of FLINs 1–6 on FLT3-ITD. With Midostaurin as a positive control, FLINs 1–6 showed nanomolar-level inhibitory activity against FLT3-ITD, with IC_50_ values ranging from 1.07 nM to 14.52 nM (Table 1; Supplementary Figure S7). It is noteworthy that FLIN-4 exhibited the most potent inhibitory effect against FLT3-ITD. The activity of FLIN-4 (IC_50_ = 1.07 ± 0.04 nM) was approximately 27 times higher than that of Midostaurin (IC_50_ = 29.64 ± 1.45 nM). In line with the molecular docking results mentioned earlier, FLIN-4 demonstrated not only the most favorable docking score but also the strongest inhibitory activity. These results further confirm the feasibility and effectiveness of the virtual screening method we utilized, and in turn, they validate the reliability of the experimental outcomes. Moreover, we extended the kinase selectivity profiling of FLIN-4 to evaluate its inhibitory activity against 60 non-target kinases. Supplementary data (Supplementary Table S3) showed that the IC_50_ values of FLIN-4 for these non-target kinases were all greater than 10 μM, thereby indicating a marked kinase selectivity for FLIN-4. These findings further reinforced the potent and specific inhibitory effect of FLIN-4 on FLT3-ITD, and due to its low off-target binding affinity, it may potentially reduce unintended side effects in clinical applications.

**TABLE 1:** The inhibitory effects of FLINs 1-6 on FLT3-ITD and MV4-11 cells.

Compounds	FLT3-ITD (IC_50_, nM)	MV4-11 (IC_50_, nM)
FLIN-1	10.24 ± 0.81	12.19 ± 0.94
FLIN-2	4.98 ± 0.35	5.54 ± 0.41
FLIN-3	2.85 ± 0.22	3.03 ± 0.27
FLIN-4	1.07 ± 0.04	1.31 ± 0.06
FLIN-5	14.52 ± 0.98	17.66 ± 1.03
FLIN-6	6.39 ± 0.47	8.28 ± 0.54
Midostaurin	29.64 ± 1.45	40.03 ± 1.62

### 3.5 MD simulation

**FIGURE 6:**
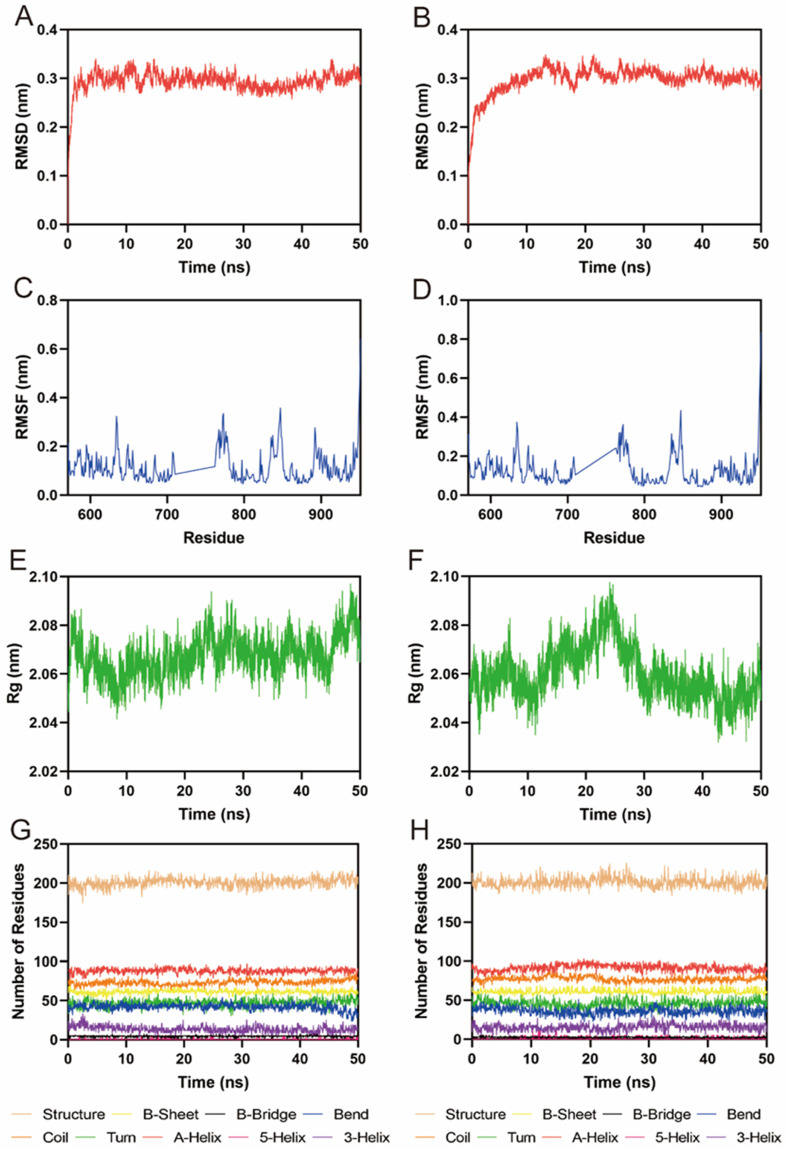
MD simulation of FLT3 in complex with FLIN-3 and FLIN-4. **(A)** RMSD of FLIN-3 in FLT3-FLIN-3 complex; **(B)** RMSD of FLIN-4 in FLT3-FLIN-4 complex; **(C, D)** RMSF of FLT3 residues in the complex of FLT3-FLIN-3 and FLT3-FLIN-4, respectively; **(E, F)** Rg of FLT3 in the complex of FLT3-FLIN-3 and FLT3-FLIN-4, respectively; **(G, H)** The secondary structures of FLT3 in the complex of FLT3-FLIN-3 and FLT3-FLIN-4, respectively.

Given the potent *in vitro* inhibitory activity of FLIN-3 and FLIN-4, we conducted a comprehensive analysis using a 50 ns molecular dynamics (MD) simulation to evaluate the binding stability of the FLT3-FLIN-3 and FLT3-FLIN-4 complexes. First, the root-mean-square deviation (RMSD) relative to the initial structure was calculated to reflect whether the complex system could maintain stability during the simulation process. In Figure 6A, the RMSD value of FLIN-3 in the FLT3-FLIN-3 complex suddenly increased but overall fluctuated within a range of approximately 0.28–0.33 nm, suggesting that FLIN-3 may stably bind to FLT3. In Figure 6B, the RMSD value of FLIN-4 in the FLT3-FLIN-4 complex first suddenly increased and then slowly increased. However, the RMSD value stabilized and fluctuated within a range of about 0.29–0.32 nm after 12 ns, suggesting that FLIN-4 may also stably bind to FLT3. However, these observations are preliminary and require experimental confirmation. Additionally, Figures 6C, D revealed the root-mean-square fluctuation (RMSF) values of FLT3 over the 50 ns simulation time to assess the stability of key residues interacting with the ligand in the complex. In the FLT3-FLIN-3 complex, the RMSF values of key residues in the active site of RAD51, including Leu616, Phe691, Glu692, Tyr693, Cys694, Tyr696, Asp698, Leu818, Arg815, and Arg834, were all less than 0.15 nm, indicating that the binding of FLT3 and FLIN-3 is relatively stable. In the FLT3-FLIN-4 complex, the RMSF values of key residues in the active site of FLT3, including Leu616, Phe691, Glu692, Tyr693, Cys694, Tyr696, Asp698, Leu818, Arg815, and Arg834, were all less than 0.22 nm, indicating that the binding of FLT3 and FLIN-4 is relatively stable. These findings, while suggestive of stability, await experimental validation for confirmation. Subsequently, Figures 6E, F showed that the Rg value fluctuations in the FLT3-FLIN-3 complex and the FLT3-FLIN-4 complex were all less than 0.06 nm, indicating that the proteins maintained their structural compactness during the simulation process. Finally, the stability of the system was assessed by analyzing the fluctuations in protein secondary structures, including B-Sheet, B-Bridge, Coil, Bend, Turn, A-Helix, 5-Helix, and 3-Helix. As shown in Figures 6G, H, there were no significant fluctuations in the protein secondary structures of the FLT3-FLIN-3 complex and the FLT3-FLIN-4 complex, indicating that the structure of FLT3 remained stable throughout. These results suggest that FLIN-3 and FLIN-4 may stably bind to the active site of FLT3 throughout the MD simulation process.

### 3.6 *In vitro* anti-proliferation assay

Owing to the pivotal role of FLT3 in the progression of acute myeloid leukemia, we conducted further investigations into the cellular anti-proliferative effects of FLINs 1–6. Human acute myeloid leukemia cells (MV4-11) were treated with various concentrations of FLINs 1–6 and Midostaurin. After a 72-h treatment, the anti-proliferative activity of FLINs 1–6 against MV4-11 cells ranged from 1.31 nM to 17.66 nM (Table 1; Supplementary Figure S8). Among them, FLIN-4 exhibited the highest inhibitory effect against MV4-11 cells (IC_50_ = 1.31 ± 0.06 nM), which was significantly stronger than Midostaurin (IC_50_ = 40.03 ± 1.62 nM). Overall, these results indicate that FLIN-4 demonstrated potent inhibitory activity against human acute myeloid leukemia cells *in vitro*, with a markedly higher inhibition rate compared to its positive control.

Subsequently, to further validate the specificity of FLIN-4 for cells expressing FLT3-ITD, we conducted MTT assays to assess the specificity and cytotoxicity of FLIN-4 within the concentration range of 2–64 μM on NB4 cells (harboring FLT3 wild-type) and COLO 829BL cells (representing normal hematopoietic cells). The analysis results indicated (Supplementary Figure S9), that within the tested concentration range of 2–64 μM, FLIN-4 did not induce any significant changes in the survival rates of NB4 or COLO 829BL cells. Notably, the cell survival rates of both cell lines remained relatively stable across the entire concentration gradient of FLIN-4, suggesting a minimal cytotoxic profile for FLIN-4 within this specific concentration range. This observation implies a high degree of selectivity of FLIN-4 for FLT3-ITD positive leukemia cells, with minimal off-target effects on normal hematopoietic cells. These findings support the specificity of FLIN-4 for FLT3-ITD expressing cells and suggest a favorable safety profile.

### 3.7 Inhibition of FLT3 signaling pathway by FLIN-4 in MV4-11 cells

**FIGURE 7:**
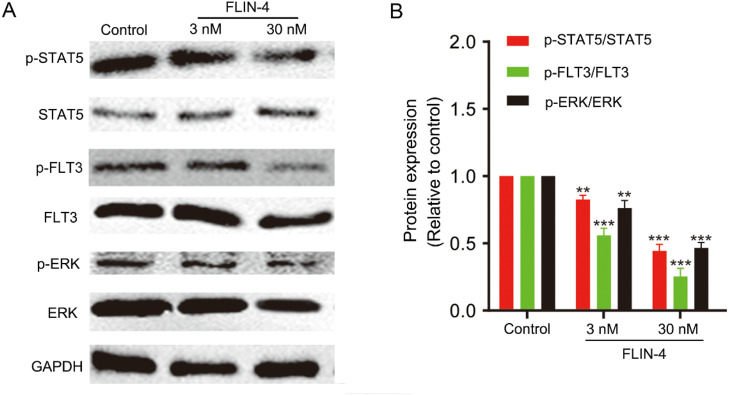
FLIN-4 inhibits FLT3 autophosphorylation and the phosphorylation of downstream signaling effectors in MV4-11 cells. **(A)** Expression of p-STAT5, STAT5, p-FLT3, FLT3, p-ERK, and ERK in MV4-11 cells treated with different concentrations of FLIN-4 for 4 h. **(B)** Quantification of the results for p-STAT5/STAT5, p-FLT3/FLT3, and p-ERK/ERK in control group and FLIN-4 treated cells at different concentrations. **p < 0.01, ***p < 0.001 compared to the control group. Results are expressed as mean ± SD, n = 3.

To elucidate the impact of FLIN-4 on the FLT3 signaling cascade, we conducted a Western blot analysis in MV4-11 cells to evaluate the relative expression levels of pivotal phosphorylated proteins. The Western blot analysis revealed a marked reduction in the expression levels of p-STAT5/STAT5, p-FLT3/FLT3, and p-ERK/ERK in a concentration-dependent manner with increasing doses of FLIN-4 (Figure 7A). Quantitative analysis corroborated these findings, demonstrating a significant diminution in the expression levels of the aforementioned phosphorylated proteins upon treatment with 3 nM FLIN-4, with an exacerbated decrease observed at a concentration of 30 nM (Figure 7B). These findings suggest that FLIN-4 exerts a potent inhibitory effect on the phosphorylation of STAT5, FLT3, and ERK. Consequently, these results highlight the potent inhibitory action of FLIN-4 on FLT3 signaling, reinforcing its potential as a therapeutic agent for targeting this pathway.

## 4 Discussion

AML is a prevalent hematological malignancy, with treatment outcomes often falling short of expectations, particularly for patients harboring FLT3 mutations (Daver et al., 2019; Estey, 2018). The FLT3 mutation represents a significant therapeutic target in AML due to its pivotal role in the initiation and progression of the disease (Kindler et al., 2010). Inhibitors targeting FLT3 can block its activity, effectively suppressing the proliferation of AML cells with FLT3 mutations (Negotei et al., 2023). These inhibitors not only aid in improving patient outcomes but also provide novel treatment options for those who cannot tolerate conventional chemotherapy.

In this study, we identified six small molecule compounds (FLINs 1–6) with potential inhibitory effects on FLT3-ITD through a virtual screening strategy. Molecular docking analysis predicted the binding modes of FLINs 1–6 with FLT3, revealing their ability to stably bind within the active site of enzyme. Notably, FLIN-4 demonstrated the strongest binding free energy to FLT3, which may correlate with its high inhibitory activity observed in subsequent experiments. Enzyme inhibition assays showed that FLINs 1–6 all exhibited nanomolar-level inhibitory activity, significantly surpassing that of the positive control drug Midostaurin. These results suggest that FLINs 1–6 could be effective inhibitors of FLT3-ITD, with FLIN-4 showing particularly prominent inhibitory activity, indicating a more pronounced suppressive effect on FLT3-ITD at the molecular level. To further elucidate the binding mechanisms of Gilteritinib and Midostaurin relative to FLIN4, an in-depth analysis of their binding modes with FLT3 was conducted. As depicted in Supplementary Figure S10A, both Gilteritinib and FLIN4 are capable of forming hydrogen bond interactions with residues Glu692, Cys694, and Arg834 within the FLT3 protein. Additionally, both compounds can engage in hydrophobic interactions with residues Tyr696, Leu818, and Tyr693 within the FLT3 protein. Furthermore, in comparison to Gilteritinib, FLIN4 is also capable of forming additional hydrogen bonds with residues Leu818 and Asp698 within the FLT3 protein. Consequently, FLIN4 may exhibit stronger interactions with the FLT3 protein than Gilteritinib. Furthermore, as illustrated in Supplementary Figure S10B, Midostaurin and FLIN4 can both form hydrogen bond interactions with residues Glu692 and Cys694 within the FLT3 protein. In comparison to Midostaurin, FLIN4 can also form additional hydrogen bonds with residues Arg834, Leu818, and Asp698 within the FLT3 protein. Therefore, FLIN4 may exhibit stronger interactions with the FLT3 protein than Midostaurin. Additionally, to explore the potential activity of FLIN4 against pan-resistant mutations such as F691L, an in-depth analysis of the binding mode of FLIN4 with the FLT3-F691L mutant was conducted. FLIN4 can form hydrogen bond interactions with residues Glu692, Cys694, Arg834, Leu818, and Asp698 within the FLT3-F691L mutant (Supplementary Figure S11). Moreover, FLIN4 can also engage in hydrophobic interactions with residues Tyr696, Leu818, and Tyr693 within the FLT3-F691L mutant. Therefore, FLIN4 may still possess strong potential activity against the FLT3-F691L mutant.

Cellular assays with human acute myeloid leukemia cells (MV4-11) further confirmed the potential of FLINs 1–6 as FLT3-ITD inhibitors. FLIN-4 had the lowest IC_50_ value, indicating its high efficacy in inhibiting cellular proliferation. Consistent with the molecular docking and enzyme inhibition assay results, these findings further support the potential of FLIN-4 as an FLT3-ITD inhibitor. Moreover, MD simulations were conducted to evaluate the stability of the FLIN-4 and FLT3 interaction. The MD simulations confirmed the stable binding of FLIN-4 to the active binding site of FLT3, providing further evidence of the robustness of this interaction. However, it is crucial to acknowledge that these results are based on computational models and have not been experimentally verified. Future work should include structural validation to confirm the stability and binding mode of these complexes.

FLT3 mutations are ubiquitous in acute myeloid leukemia (AML) and are significantly correlated with poor prognostic outcomes (Kennedy and Smith, 2020). The initial efficacy of FLT3 inhibitors is compromised by the emergence of resistance, which is classified into primary and secondary forms (Eguchi et al., 2020). Primary resistance is linked to intrinsic tumor cell factors, including the overexpression of CYP3A4 and the activation of FLT3 ligand; secondary resistance is associated with *de novo* FLT3 mutations and the aberrant activation of downstream signaling pathways (Ruglioni et al., 2024). In this study, we have identified a novel small molecule inhibitor, FLIN-4, which exhibits pronounced and selective inhibition of FLT3-ITD, suggesting a potential advantage in combating resistance. FLIN-4 demonstrates superior inhibitory activity against FLT3-ITD compared to the positive control Midostaurin, implying its capacity to overcome resistance driven by aberrant FLT3 kinase activity. In cellular assays, FLIN-4 significantly impedes the proliferation of the MV4-11 AML cell line, which carries an exclusive FLT3-ITD mutation, while exerting minimal toxicity on cell lines with FLT3-wildtype and normal hematopoietic cells. This selectivity indicates a reduced impact on normal cellular elements and a potential reduction in resistance associated with drug toxicity. Moreover, FLIN-4 substantially inhibits FLT3 autophosphorylation and the phosphorylation of downstream key target proteins in MV4-11 cells, suggesting its ability to interrupt critical nodes within the FLT3 signaling axis, thereby disrupting the proliferative and survival signaling of leukemia cells and potentially overcoming resistance arising from the aberrant activation of downstream pathways. To further elucidate the role of FLIN-4 in resistance mechanisms, we intend to conduct a series of experiments in forthcoming studies, aiming to delineate its efficacy in surmounting drug resistance.

Our study confirms that the targeted inhibition of FLT3-ITD significantly reduces the proliferation of acute myeloid leukemia cells. The discovery of FLIN-4 provides a novel chemical entity for the development of FLT3-ITD inhibitors, which may have significant implications for the treatment of AML. Future research can further explore the pharmacological properties of FLIN-4, including its pharmacokinetics and toxicology *in vivo*, as well as how to further enhance its inhibitory activity and selectivity through structural optimization.

## 5 Conclusion

The FLT3-ITD mutation plays a significant role in the pathogenesis of acute myeloid leukemia (AML) and represents a potential therapeutic target for the disease. In this study, we successfully identified a novel, potent small molecule inhibitor, FLIN-4, which exhibits effective and selective inhibitory activity against FLT3-ITD (IC_50_ = 1.07 ± 0.04 nM). Furthermore, *in vitro* anti-proliferation assays demonstrated that FLIN-4 can suppress the growth of acute myeloid leukemia cells. Thus, FLIN-4 holds promise for further development as a new cancer therapeutic agent. Notably, the consistency between the biological assay results and the docking scores validated the use of structure-based virtual screening for predicting the activity of lead compounds.

In summary, we reported a potential novel FLT3 inhibitor. This discovery provides a solid foundation for the development of therapies to treat acute myeloid leukemia.

## Data Availability

The original contributions presented in the study are included in the article/Supplementary Material, further inquiries can be directed to the corresponding authors.

## References

[B1] Abbas H. A. Alfayez M. Kadia T. Ravandi-Kashani F. Daver N. (2019). Midostaurin in acute myeloid leukemia: an evidence-based review and patient selection. Cancer Manag. Res. 11, 8817–8828. 10.2147/CMAR.S177894 31632141 PMC6782026

[B2] Anderson L. J. Girguis M. Kim E. Shewale J. Braunlin M. Werther W. (2024). A temporal and multinational assessment of acute myeloid leukemia (AML) cancer incidence, survival, and disease burden. Leuk. Lymphoma 65 (10), 1482–1492. 10.1080/10428194.2024.2360536 38932630

[B3] Antar A. I. Otrock Z. K. Jabbour E. Mohty M. Bazarbachi A. (2020). FLT3 inhibitors in acute myeloid leukemia: ten frequently asked questions. Leukemia 34 (3), 682–696. 10.1038/s41375-019-0694-3 31919472

[B4] Castaño-Bonilla T. Alonso-Dominguez J. M. Barragán E. Rodríguez-Veiga R. Sargas C. Gil C. (2021). Prognostic significance of FLT3-ITD length in AML patients treated with intensive regimens. Sci. Rep. 11 (1), 20745. 10.1038/s41598-021-00050-x 34671057 PMC8528825

[B5] Cortes J. Perl A. E. Döhner H. Kantarjian H. Martinelli G. Kovacsovics T. (2018). Quizartinib, an FLT3 inhibitor, as monotherapy in patients with relapsed or refractory acute myeloid leukaemia: an open-label, multicentre, single-arm, phase 2 trial. Lancet. Oncol. 19 (7), 889–903. 10.1016/S1470-2045(18)30240-7 29859851 PMC8152787

[B6] Daver N. Schlenk R. F. Russell N. H. Levis M. J. (2019). Targeting FLT3 mutations in AML: review of current knowledge and evidence. Leukemia 33 (2), 299–312. 10.1038/s41375-018-0357-9 30651634 PMC6365380

[B7] Daver N. Venugopal S. Ravandi F. (2021). FLT3 mutated acute myeloid leukemia: 2021 treatment algorithm. Blood Cancer J. 11 (5), 104. 10.1038/s41408-021-00495-3 34045454 PMC8159924

[B8] Eguchi M. Minami Y. Kuzume A. Chi S. (2020). Mechanisms underlying resistance to FLT3 inhibitors in acute myeloid leukemia. Biomedicines 8 (8), 245. 10.3390/biomedicines8080245 32722298 PMC7459983

[B9] Estey E. H. (2018). Acute myeloid leukemia: 2019 update on risk-stratification and management. Am. J. Hematol. 93 (10), 1267–1291. 10.1002/ajh.25214 30328165

[B10] Fletcher L. Joshi S. K. Traer E. (2020). Profile of quizartinib for the treatment of adult patients with relapsed/refractory FLT3-ITD-positive acute myeloid leukemia: evidence to date. Cancer Manag. Res. 12, 151–163. 10.2147/CMAR.S196568 32021432 PMC6955578

[B11] Griffith J. Black J. Faerman C. Swenson L. Wynn M. Lu F. (2004). The structural basis for autoinhibition of FLT3 by the juxtamembrane domain. Mol. Cell 13 (2), 169–178. 10.1016/s1097-2765(03)00505-7 14759363

[B12] James A. J. Smith C. C. Litzow M. Perl A. E. Altman J. K. Shepard D. (2020). Pharmacokinetic profile of Gilteritinib: a novel FLT-3 tyrosine kinase inhibitor. Clin. Pharmacokinet. 59 (10), 1273–1290. 10.1007/s40262-020-00888-w 32304015 PMC7550323

[B13] Joshi U. Bhetuwal U. Yadav S. K. Budhathoki P. Gaire S. Sharma S. (2024). Survival outcomes and prognostic factors in therapy-related acute myeloid leukemia: a seer database study, 2000-2020. Clin. Lymphoma Myeloma Leuk. 24, e827–e834.e1. 10.1016/j.clml.2024.07.002 39095252

[B14] Kantarjian H. Kadia T. DiNardo C. Daver N. Borthakur G. Jabbour E. (2021). Acute myeloid leukemia: current progress and future directions. Blood Cancer J. 11 (2), 41. 10.1038/s41408-021-00425-3 33619261 PMC7900255

[B15] Kawashima N. (2021). FLT3 inhibitors in the treatment of FLT3-mutated acute myeloid leukemia. Rinsho ketsueki Jpn. J. Clin. Hematol. 62 (8), 954–966. 10.11406/rinketsu.62.954 34497236

[B16] Kennedy V. E. Smith C. C. (2020). FLT3 mutations in acute myeloid leukemia: key concepts and emerging controversies. Front. Oncol. 10, 612880. 10.3389/fonc.2020.612880 33425766 PMC7787101

[B17] Kindler T. Lipka D. B. Fischer T. (2010). FLT3 as a therapeutic target in AML: still challenging after all these years. Blood 116 (24), 5089–5102. 10.1182/blood-2010-04-261867 20705759

[B18] Kiyoi H. Kawashima N. Ishikawa Y. (2020). FLT3 mutations in acute myeloid leukemia: therapeutic paradigm beyond inhibitor development. Cancer Sci. 111 (2), 312–322. 10.1111/cas.14274 31821677 PMC7004512

[B19] Li K. Du Y. Cai Y. Liu W. Lv Y. Huang B. (2023). Single-cell analysis reveals the chemotherapy-induced cellular reprogramming and novel therapeutic targets in relapsed/refractory acute myeloid leukemia. Leukemia 37 (2), 308–325. 10.1038/s41375-022-01789-6 36543880 PMC9898038

[B20] Marjoncu D. Andrick B. G. (2020). Gilteritinib: a novel FLT3 inhibitor for relapsed/refractory acute myeloid leukemia. J. Adv. Pract. Oncol. 11 (1), 104–108. 10.6004/jadpro.2020.11.1.7 33542854 PMC7517771

[B21] Negotei C. Colita A. Mitu I. Lupu A. R. Lapadat M. E. Popovici C. E. (2023). A review of FLT3 kinase inhibitors in AML. J. Clin. Med. 12 (20), 6429. 10.3390/jcm12206429 37892567 PMC10607239

[B22] Nogami A. Oshikawa G. Okada K. Fukutake S. Umezawa Y. Nagao T. (2015). FLT3-ITD confers resistance to the PI3K/Akt pathway inhibitors by protecting the mTOR/4EBP1/Mcl-1 pathway through STAT5 activation in acute myeloid leukemia. Oncotarget 6 (11), 9189–9205. 10.18632/oncotarget.3279 25826077 PMC4496211

[B23] Novatcheva E. D. Anouty Y. Saunders I. Mangan J. K. Goodman A. M. (2022). FMS-like tyrosine kinase 3 inhibitors for the treatment of acute myeloid leukemia. Clin. Lymphoma Myeloma Leukemia 22 (3), e161–e184. 10.1016/j.clml.2021.09.002 34649791

[B24] Ruglioni M. Crucitta S. Luculli G. I. Tancredi G. Del Giudice M. L. Mechelli S. (2024). Understanding mechanisms of resistance to FLT3 inhibitors in adult FLT3-mutated acute myeloid leukemia to guide treatment strategy. Crit. Rev. Oncol. Hematol. 201, 104424. 10.1016/j.critrevonc.2024.104424 38917943

[B25] Sadybekov A. V. Katritch V. (2023). Computational approaches streamlining drug discovery. Nature 616 (7958), 673–685. 10.1038/s41586-023-05905-z 37100941

[B26] Siegel R. L. Giaquinto A. N. Jemal A. (2024). Cancer statistics, 2024. CA Cancer J. Clin. 74 (1), 12–49. 10.3322/caac.21820 38230766

[B27] Sung P. J. Selvam M. Riedel S. S. Xie H. M. Bryant K. Manning B. (2024). FLT3 tyrosine kinase inhibition modulates PRC2 and promotes differentiation in acute myeloid leukemia. Leukemia 38 (2), 291–301. 10.1038/s41375-023-02131-4 38182819 PMC11141246

[B28] Swaminathan M. Kantarjian H. M. Levis M. Guerra V. Borthakur G. Alvarado Y. (2021). A phase I/II study of the combination of quizartinib with azacitidine or low-dose cytarabine for the treatment of patients with acute myeloid leukemia and myelodysplastic syndrome. Haematologica 106 (8), 2121–2130. 10.3324/haematol.2020.263392 33853292 PMC8327731

[B29] Takahashi S. (2011). Downstream molecular pathways of FLT3 in the pathogenesis of acute myeloid leukemia: biology and therapeutic implications. J. Hematol. and Oncol. 4, 13. 10.1186/1756-8722-4-13 21453545 PMC3076284

[B30] Takahashi T. Usuki K. Matsue K. Ohno H. Sakura T. Imanaka R. (2019). Efficacy and safety of quizartinib in Japanese patients with FLT3-ITD positive relapsed or refractory acute myeloid leukemia in an open-label, phase 2 study. Int. J. Hematol. 110 (6), 665–674. 10.1007/s12185-019-02727-6 31473943

[B31] Tang W. Chen Z. Zhang W. Cheng Y. Zhang B. Wu F. (2020). The mechanisms of sorafenib resistance in hepatocellular carcinoma: theoretical basis and therapeutic aspects. Signal Transduct. Target. Ther. 5 (1), 87. 10.1038/s41392-020-0187-x 32532960 PMC7292831

[B32] Usuki K. Handa H. Choi I. Yamauchi T. Iida H. Hata T. (2019). Safety and pharmacokinetics of quizartinib in Japanese patients with relapsed or refractory acute myeloid leukemia in a phase 1 study. Int. J. Hematol. 110 (6), 654–664. 10.1007/s12185-019-02709-8 31359361

[B33] van der Wel T. Hilhorst R. den Dulk H. van den Hooven T. Prins N. M. Wijnakker J. (2020). Chemical genetics strategy to profile kinase target engagement reveals role of FES in neutrophil phagocytosis. Nat. Commun. 11 (1), 3216. 10.1038/s41467-020-17027-5 32587248 PMC7316778

[B34] Wei T.-H. Wang Z.-X. Lu M.-Y. Xu Y.-J. Yang J. Ni X.-F. (2024). Discovery of SILA-123 as a highly potent FLT3 inhibitor for the treatment of acute myeloid leukemia with various FLT3 mutations. J. Med. Chem. 67, 21752–21780. 10.1021/acs.jmedchem.4c00529 39258312

[B35] Young J. A. Pallas C. R. Knovich M. A. (2021). Transplant-associated thrombotic microangiopathy: theoretical considerations and a practical approach to an unrefined diagnosis. Bone Marrow Transplant. 56 (8), 1805–1817. 10.1038/s41409-021-01283-0 33875812 PMC8338557

[B36] Yuan W. Zhang S. Zhu H. (2022). Advances in clinical studies of FLT3 inhibitors in acute myeloid leukemia. Zhejiang Da Xue Xue Bao Yi Xue Ban 51 (4), 507–514. 10.3724/zdxbyxb-2022-0090 37202100 PMC10264991

[B37] Zhao J. C. Agarwal S. Ahmad H. Amin K. Bewersdorf J. P. Zeidan A. M. (2022). A review of FLT3 inhibitors in acute myeloid leukemia. Blood Rev. 52, 100905. 10.1016/j.blre.2021.100905 34774343 PMC9846716

[B38] Zhou Y. Zou Y. Yang M. Mei S. Liu X. Han H. (2022). Highly potent, selective, biostable, and cell-permeable cyclic d-peptide for dual-targeting therapy of lung cancer. J. Am. Chem. Soc. 144 (16), 7117–7128. 10.1021/jacs.1c12075 35417174

