# Supplementary material for: Discovery of novel and highly potent small molecule inhibitors targeting FLT3-ITD for the treatment of acute myeloid leukemia using structure-based virtual screening and biological evaluation
Source: Front Pharmacol. 2025 Feb 3;16:1511257. doi: 10.3389/fphar.2025.1511257 (PMC11830721; doi:10.3389/fphar.2025.1511257)
Supplement: Supplementary file 1 [file DataSheet1.docx]

**Supplementary Material**

**Discovery of Novel and Highly Potent Small Molecule Inhibitors Targeting FLT3-ITD for the Treatment of Acute Myeloid Leukemia Using Structure-Based Virtual Screening and Biological Evaluation**

1. **Supplementary** **Figures and Tables**
   1. **Supplementary figures**

| Rank | Retention time (min) | Area | Conc |
| --- | --- | --- | --- |
| 1 | 7.635 | 78969 | 0.8835 |
| 2 | 8.078 | 41225 | 0.4610 |
| 3 | 8.248 | 8823097 | 98.6560 |
| Total |  | 8943291 | 100 |

**Supplementary Figure S1.** The HPLC chromatogram of the synthesized compound FLIN-1.

| Rank | Retention time (min) | Area | Conc |
| --- | --- | --- | --- |
| 1 | 8.472 | 5537.246 | 0.0925 |
| 2 | 9.388 | 63081.48 | 1.0538 |
| 3 | 9.688 | 5876901.5 | 98.1729 |
| 4 | 9.688 | 40758.227 | 0.6808 |
| Total |  | 5986278.453 | 100 |

**Supplementary Figure S2.** The HPLC chromatogram of the synthesized compound FLIN-2.

| Rank | Retention time (min) | Area | Conc |
| --- | --- | --- | --- |
| 1 | 6.988 | 19421.551 | 0.3878 |
| 2 | 7.200 | 6955.940 | 0.1389 |
| 3 | 9.725 | 1659.866 | 0.0331 |
| 4 | 10.198 | 4936851.00 | 98.5844 |
| 5 | 12.050 | 3232.231 | 0.0645 |
| 6 | 12.438 | 39624.602 | 0.7913 |
| Total |  | 5007745.19 | 100 |

**Supplementary Figure S3.** The HPLC chromatogram of the synthesized compound FLIN-3.

| Rank | Retention time (min) | Area | Conc |
| --- | --- | --- | --- |
| 1 | 8.903 | 7023 | 0.4178 |
| 2 | 9.220 | 13609 | 0.8097 |
| 3 | 9.728 | 1649122 | 98.12 |
| 4 | 9.987 | 5106 | 0.3038 |
| 5 | 10.989 | 5858 | 0.3487 |
| Total |  | 1680718 | 100 |

**Supplementary Figure S4.** The HPLC chromatogram of the synthesized compound FLIN-4.

| Rank | Retention time (min) | Area | Conc |
| --- | --- | --- | --- |
| 1 | 6.873 | 11743 | 0.1197 |
| 2 | 8.063 | 105145 | 1.063 |
| 3 | 8.508 | 42540 | 0.4335 |
| 4 | 8.725 | 9626351 | 98.09 |
| 5 | 9.217 | 28017 | 0.2855 |
| Total |  | 9813769 | 100 |

**Supplementary Figure S5.** The HPLC chromatogram of the synthesized compound FLIN-5.

| Rank | Retention time (min) | Area | Conc |
| --- | --- | --- | --- |
| 1 | 12.238 | 25820 | 0.1706 |
| 2 | 12.407 | 153865 | 1.016 |
| 3 | 12.642 | 14862613 | 98.19 |
| 4 | 12.903 | 94536 | 0.6245 |
| Total |  | 15136834 | 100 |

**Supplementary Figure S6.** The HPLC chromatogram of the synthesized compound FLIN-6.





**Supplementary Figure S7.** The inhibitory effects of the FLINs 1-6 on FLT3-ITD kinase activity. The data presented are expressed as the mean ± SD derived from triplicate independent experiments.


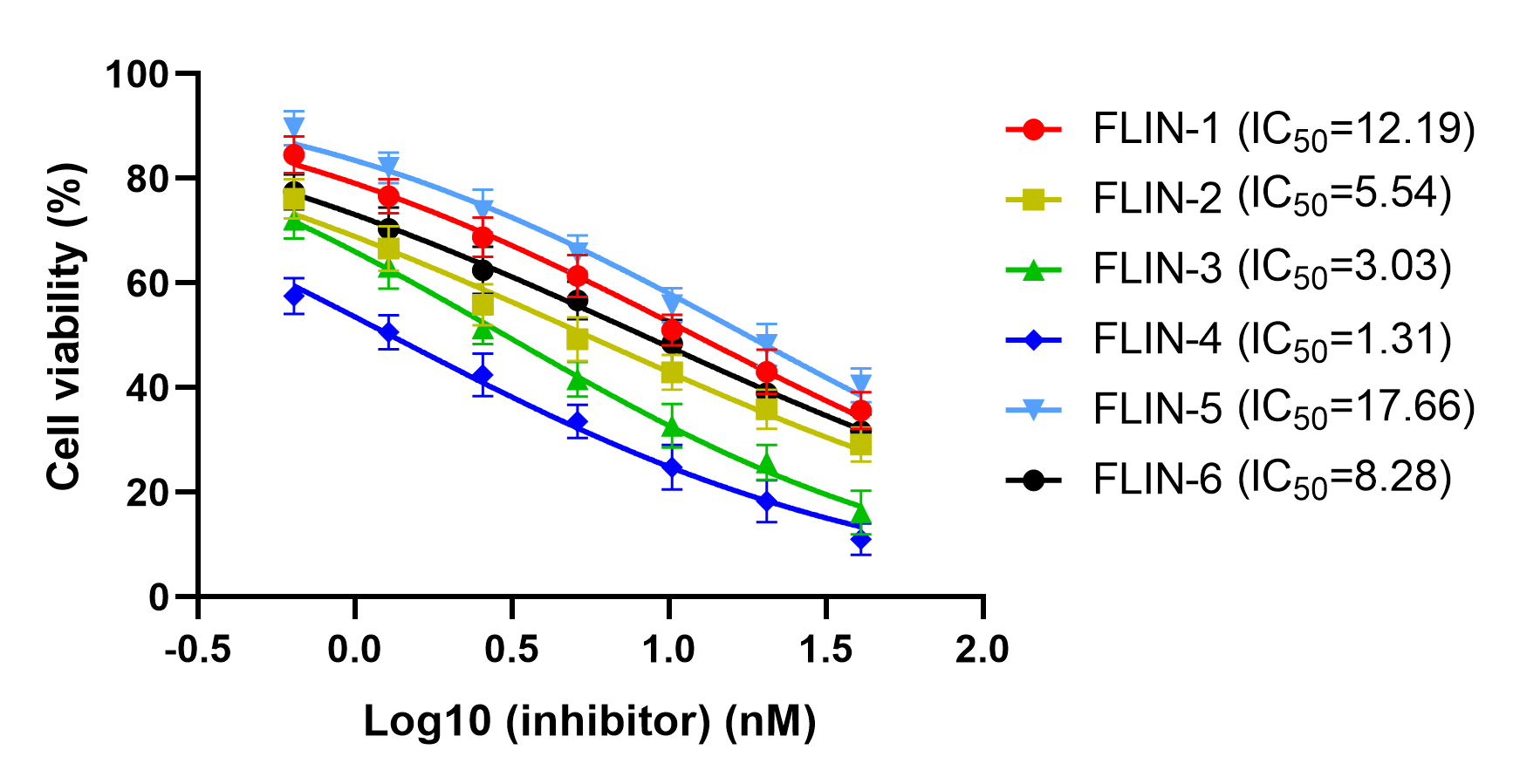


**Supplementary Figure S8.** Influence of inhibitors (FLINs 1-6) on the antiproliferative activity against the MV4-11 cell line. The data are expressed as the mean ± SD derived from triplicate independent experiments.





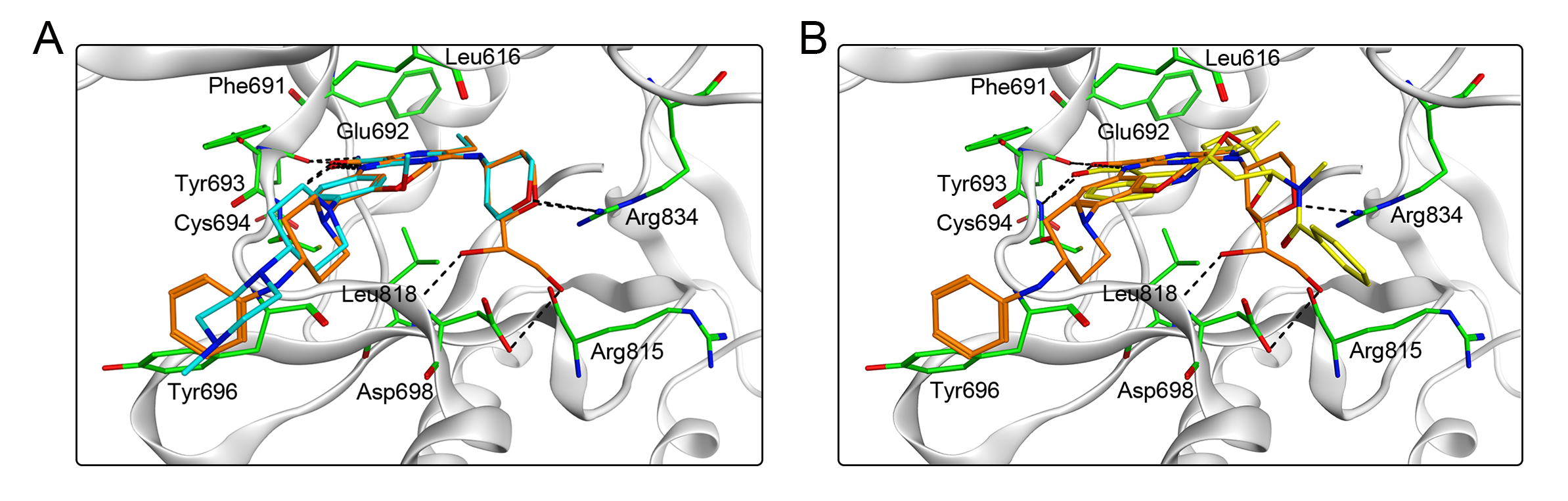
**Supplementary Figure S9.** Viability of NB4 cells (FLT3 wild-type) and Colo 829BL cells (normal hematopoietic) following treatment with FLIN-4 at concentrations from 2 to 64 μM as determined by MTT assay. Values represent the mean ± SD of three independent experiments.

**Supplementary Figure S10.** **(A)** Binding modes of Gilteritinib and FLIN4 with FLT3. **(B)** Binding modes of Midostaurin and FLIN4 with FLT3. Gilteritinib, cyan; Midostaurin, yellow; FLIN4, orange. Residues on the FLT3 binding surface are represented as green sticks. Hydrogen bonds are indicated by black dashed lines.


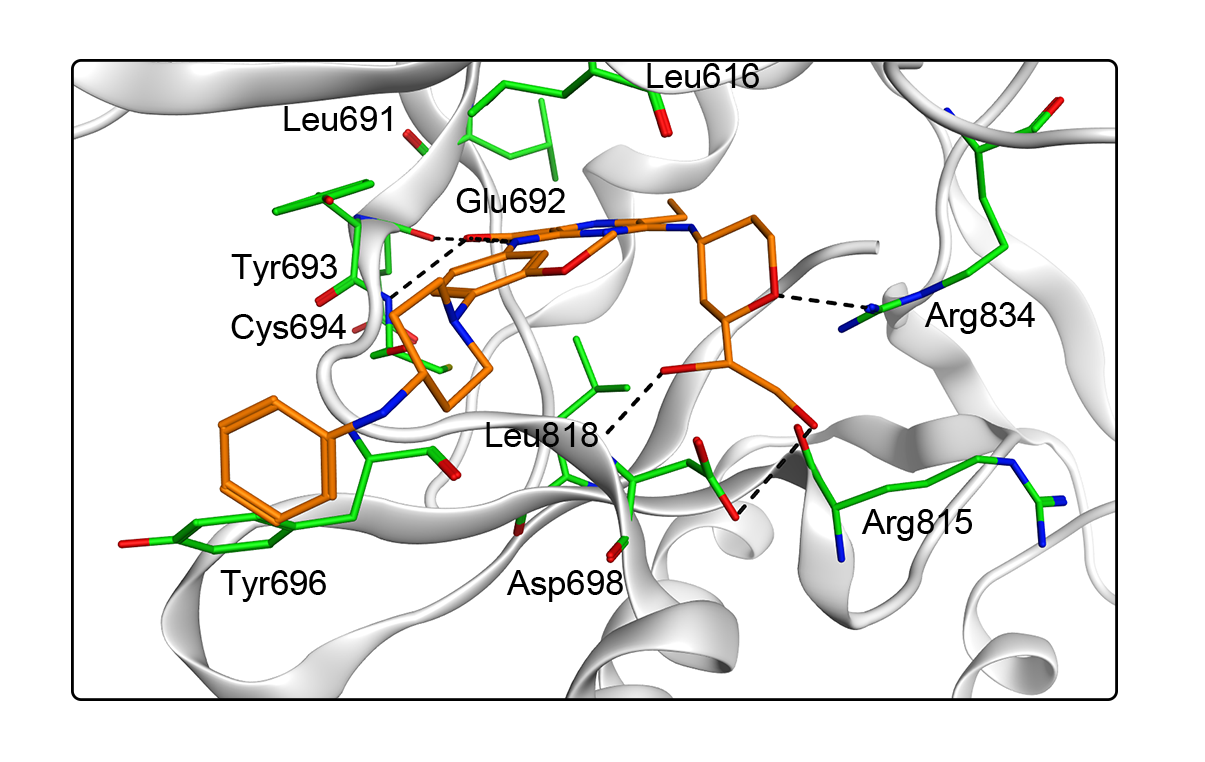


**Supplementary Figure S11.** The binding mode of FLIN4 with the FLT3-F691L mutant. FLIN4, orange. Residues on the binding surface of the FLT3-F691L mutant are represented as green sticks. Hydrogen bonds are indicated by black dashed lines.

- 1. **Supplementary tables**

**Supplementary Table S1.** Lot numbers of the identified compounds.

| **Compounds** | **Lot NO** |
| --- | --- |
| FLIN-1 | P240120-XT1 |
| FLIN-2 | P240120-XT2 |
| FLIN-3 | P240120-XT3 |
| FLIN-4 | P240120-XT4 |
| FLIN-5 | P240120-XT5 |
| FLIN-6 | P240120-XT6 |

**Supplementary Table S2.** Affinity evaluation of FLIN-4 binding to FLT3-ITD.

| **Compounds** | **FLT3-ITD (*K*_d_, nM)** |
| --- | --- |
| FLIN-1 | 8.74 ± 0.52 |
| FLIN-2 | 3.62 ± 0.41 |
| FLIN-3 | 2.19 ± 0.33 |
| FLIN-4 | 0.95 ± 0.06 |
| FLIN-5 | 12.47 ± 1.05 |
| FLIN-6 | 5.26 ± 0.42 |
| Midostaurin | 17.08 ± 3.14 |

**Supplementary Table S3.** Selectivity testing of FLIN-4 on a panel of kinases.

| **Target** | **IC_50_ (μM)** | **Target** | **IC_50_ (μM)** | **Target** | **IC_50_ (μM)** |
| --- | --- | --- | --- | --- | --- |
| ABL1 | > 10 | FES | > 10 | LTK | > 10 |
| ABL2 | > 10 | FGFR1 | > 10 | LYN | > 10 |
| AXL | > 10 | FGFR2 | > 10 | MERTK | > 10 |
| BLK | > 10 | FGFR3 | > 10 | MET | > 10 |
| BMX | > 10 | FGFR4 | > 10 | MST1R | > 10 |
| BTK | > 10 | FGR | > 10 | MUSK | > 10 |
| CSF1R | > 10 | FRK | > 10 | NTRK1 | > 10 |
| PLK1 | > 10 | FYN | > 10 | NTRK2 | > 10 |
| DDR1 | > 10 | PIM1 | > 10 | NTRK3 | > 10 |
| DDR2 | > 10 | RAF1 | > 10 | PDGFRA | > 10 |
| ALK | > 10 | ROS1 | > 10 | PDGFRB | > 10 |
| EPHA1 | > 10 | ZAK | > 10 | PTK2 | > 10 |
| EPHA2 | > 10 | TYRO3 | > 10 | CDK1 | > 10 |
| EPHA3 | > 10 | YES1 | > 10 | CDK2 | > 10 |
| EPHA4 | > 10 | ZAP70 | > 10 | EPHB3 | > 10 |
| EPHA5 | > 10 | HCK | > 10 | EPHB4 | > 10 |
| EPHA6 | > 10 | IGF1R | > 10 | ERBB2 | > 10 |
| EPHA8 | > 10 | INSRR | > 10 | JAK2 | > 10 |
| EPHB1 | > 10 | ITK | > 10 | JAK3 | > 10 |
| EPHB2 | > 10 | JAK1 | > 10 | KIT | > 10 |
